# Supplementary material for: Screening and Immune Efficacy Evaluation of Antigens with Protection Against Feline Calicivirus
Source: Vaccines (Basel). 2024 Oct 24;12(11):1205. doi: 10.3390/vaccines12111205 (PMC11598032; doi:10.3390/vaccines12111205)
Supplement: Supplementary file 1 [file vaccines-12-01205-s001.zip › vaccines-3199021-supplementary.pdf]

Supplementary Materials:

Figure S1

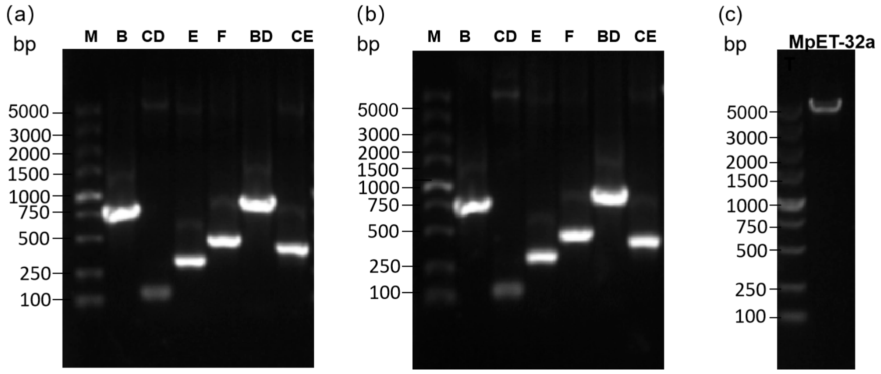

Figure. S1 Amplification of DL39 and FB-NJ-13 recombinant fragments and enzyme digestion of carrier plasmid. (a) Amplification of DL39 recombinant fragments. (b) Amplification of FB-NJ-13 recombinant fragment. (c) Plasmid enzyme cleavage.

Figure S2

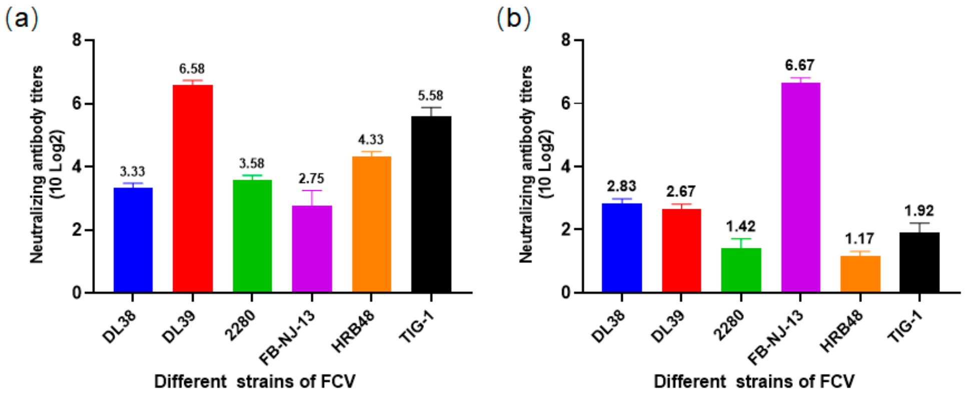

Fig. S2 Results of cross-neutralization test. (a) Cross-neutralization test of feline sera prepared against DL39. (b) Cross-neutralization test of feline sera prepared against FB-NJ-13.

Figure S3

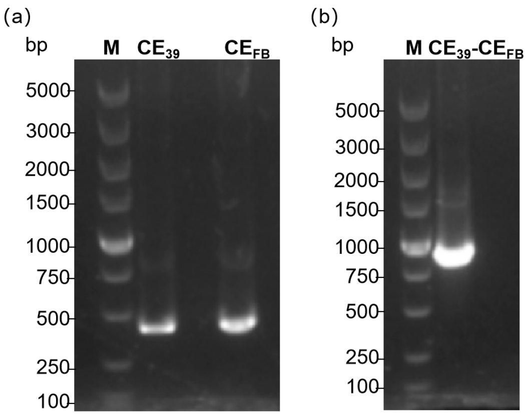

Figure S3. Construction of the CE$_{39}$-CE$_{FB}$ plasmid. (a) Amplification of CE$_{39}$ and CE$_{FB}$. (b) Fusion PCR of CE$_{39}$-CE$_{FB}$.

Table S1

Table S1. Amplification primers for each segment of the VP1

Primer name	Primer sequence (5'-3')
DL39-B-F	gccatggctgatatcggatccGCTGATGGGGACGGTTCC
DL39-B-R	gtggtggtggtggtgctcgagGCTGATGCTGATAGTGATTGGTCT
DL39-CD-F	gccatggctgatatcggatccCAAAGTAATAGTGCAAGATTGGGAA
DL39-CD-R	gtggtggtggtggtgctcgagAATTGTTGTATCGGGCCAACC
DL39-E-F	gccatggctgatatcggatccCCTGGGGAATTAACCCCGG
DL39-E-R	gtggtggtggtggtgctcgagAACCTCTTTGTTAACATGATTGTCCT
DL39-F-F	gccatggctgatatcggatccCAAACATCAGATGTTACGCTAGCA
DL39-F-R	gtggtggtggtggtgctcgagTAGTTTTGTCATTGAGCTCCTAATGTT
FB-NJ-13-B-F	gccatggctgatatcggatccTCTGACGATGGTTCAATTACAACG
FB-NJ-13-B-R	gtggtggtggtggtgctcgagACTAATGGTTATTGTCATTGGTCTAAATC
FB-NJ-13-CD-F	gccatggctgatatcggatccCAAAAGCAATCAGCAAAATTGG
FB-NJ-13-CD-R	gtggtggtggtggtgctcgagAATGGTTGTGTCTGGCCAACC
FB-NJ-13-E-F	gccatggctgatatcggatccGATAGCAATCTGATTCCTGCAGG
FB-NJ-13-E-R	gtggtggtggtggtgctcgagAACTTGTAAATTCACATGATTATCCTGG
FB-NJ-13-F-F	gccatggctgatatcggatccCAAACATCTGAAGTTACTCTAGCAATGC
FB-NJ-13-F-R	gtggtggtggtggtgctcgagTAATTTGGTCATTGAGCTCCTAATG

Note: Underline represents the cleavage site sequence

Table S2

Table S2. Amplification primers for CE$_{39}$-CE$_{FB}$ recombinant protein

Primer name	Length	Primer sequence (5'-3')
DL39+FB-NJ-CE-F1	393 bp	GAGGTTGGCGGCGGCGGCTCGCAAAAGCAATCAGCAAAATTGG
FB-NJ-13-E-R		gtggtggtggtggtgctcgagAACTTGTAAATTCACATGATTATCCTGG
DL39-CD-F	393 bp	gccatggctgatatcggatccCAAAGTAATAGTGCAAGATTGGGAA
DL39+FB-NJ-CE-R1		GCTTTTGCGAGCCGCCGCCGCCAACCTCTTTGTTAACATGA

Note: Underline represents the cleavage site sequence

Table S3

Table S3. Animal clinical symptom rating table

Clinical symptoms	Symptom severity	Score
Oral ulcer	Severe (ulceration, more than 0.3cm in diameter or more than 2 ulcers)	2
	Slight (less than 0.3cm in diameter or 2 or less on ulcers)	1
	None	0
Eye and nose discharge	Severe (eyelids are covered, affecting normal activities)	2
	Mild (less discharge, does not affect normal activities)	1
	None	0
Body temperature	> 40°C or < 37.5°C	2
	37.5°C-38°C or 39.0°C-40°C	1
	38.0°C-39.0°C	0
Body weight	Reduce by more than 10%	2
	Reduce by less than 10%	1
	Continue to increase	0
Mental state	Depression and loss of appetite	2
	Depression or loss of appetite	1
	Normal	0
Cough and other symptoms	Severe (persistent cough, difficulty breathing)	2
	Mild (intermittent cough)	1
	None	0
Death	Yes	5
	No	0

Table S4

Table S4. Optimal expression conditions for each recombinant protein

**Proteins**	**Expression condition**		
	**Final concentration of IPTG (nmol/μL)**	**Temperature (°C)**	**Time (h)**
B_{39}	0.25	16	15
CD_{39}	0.25	16	15
E_{39}	0.5	16	18
F_{39}	0.4	18	8

BD_{39}	0.25	16	18
CE_{39}	0.25	16	16
B_{FB}	0.25	16	16
CD_{FB}	0.25	16	16
E_{FB}	0.5	16	20
F_{FB}	0.4	18	10
BD_{FB}	0.25	16	18
CE_{FB}	0.25	16	16

Table S5

Table S5. Concentrations of purified recombinant proteins

Proteins	Concentration (ng/μL)	Proteins	Concentration (ng/μL)
B_{39}	426	B_{FB}	453
CD_{39}	512	CD_{FB}	566
E_{39}	608	E_{FB}	502
F_{39}	589	F_{FB}	543
BD_{39}	416	BD_{FB}	408
CE_{39}	626	CE_{FB}	735
